# Knockout of KDM3A in MDA-MB-231 breast cancer cells inhibits tumor malignancy and promotes apoptosis

**DOI:** 10.1007/s10735-023-10178-x

**Published:** 2024-01-02

**Authors:** Yuanxing Han, Nueryemu Maimaiti, Yue Sun, Juan Yao

**Affiliations:** 1 The First Affiliated Hospital of Xinjiang Medical University, Urumqi, Xinjiang 830000 China; 2 Imaging Center of the First Affiliated Hospital of Xinjiang Medical University, Urumqi, Xinjiang 830000 China

**Keywords:** KDM3A, Lysine demethylase 3A, Breast cancer, Metastases

## Abstract

The histone lysine demethylase 3 A (KDM3A) is vital for the regulation of cancer physiology and pathophysiology. The purpose of this study was to investigate the effect of KDM3A expression with triple-negative breast cancer (TNBC) invasion and metastasis. In our results, knockout of KDM3A in TNBC MDA-MB-231 cells promoted apoptosis and inhibited the proliferation, invasion and metastasis of MDA-MB-231 cells. In addition, we found that in vivo experiments indicated that the growth, invasion and metastasis of metastatic neoplasms were significantly inhibited by knockout of KDM3A in a TNBC metastasis model. These findings suggest that KDM3A may be a potential therapeutic target for the treatment and prevention of TNBC, providing a critical theoretical basis for the effective prevention or treatment of breast cancer disease.

## Introduction

The breast cancer (BC) has emerged as the most prevalent form of malignancy worldwide. surpassing lung cancer, with an annual incidence of 2.26 million cases (Sung et al. 2021). According to the recent research, the overall survival rate for primary BC in 5 years has been nearly 90%. Nevertheless, 33% of BC patients appear non-nodal distant metastases that shorten overall survival rate of 5 years down to 23% (Siegel et al. 2020). Despite the advancements in medical technology and early detection techniques, the global incidence and mortality rates of BC continue to rise, although a decline has been observed in developed nations (Balazy et al. 2019; Feng et al. 2019). Regrettably, BC patients continue to face unfavorable prognoses due to the high occurrence of metastatic, invasiveness and recurrence (Coleman et al. 2020a; Mariotto et al. 2017). Notably, Clinical research revealed that approximately 80% of advanced BC patients experience bone metastasis, resulting in complications related to bone health (R. E. Coleman et al. 2020b; Riggio et al. 2021; Yao et al. 2019). Despite a deceleration in the decline of BC mortality, it remains the primary cause of death among BC patients (Bongiovanni et al. 2021; Cao et al. 2021), and effective treatment options remain insufficient (Invernizzi et al. 2020). Recent study has found that a new type of circRNA can induce BC metastasis by enhancing epithelial mesenchymal transition (Liu et al. 2022). Furthermore, some scholars put forward myoepithelial cell layer focal abnormal degeneration as the most likely trigger for breast cancer invasion (Man et al. 2023). Nevertheless, biological regulatory functions of The histone lysine demethylase 3 A (KDM3A) in the accentuation of bone metastasis of BC has not been elucidated yet.

KDM3A, a critical member of the H3K9me2/1 demethylase family, exhibits diverse regulatory functions in association with target genes. Its involvement in the core regulatory mechanisms governing the proliferation and differentiation of neural stem cells, hematopoietic stem cells (Ikeda et al. 2018), and embryonic stem cells underscores its significance in tumorigenesis (Lee et al. 2019), development (Wang et al. 2019) and metastasis (Li et al. 2017). Suppression of KDM3A has been shown to inhibit cell proliferation (Qin et al. 2017; Wilson et al. 2017), while further depletion of KDM3A demonstrates inhibitory effects on cell proliferation, migration, and invasion (Ikeda et al. 2018; Ramadoss et al. 2017a; Sun et al. 2019). All the above findings indicated that KDM3A were essential in progression of cancers and are ideal biomarkers of predicting prognosis and new therapeutic targets. However, little is discovered around the biological mechanism of KDM3A that wound accentuate the ability of invasion of BC cells in progression.

In this study, RNA interference technology was employed to effectively suppress the expression of human BC epithelial cell line MDA-MB-231 followed by the establishment of a mouse model of BC bone metastasis. Both in vitro and in vivo investigations were conducted to comprehensively elucidate the impact of KDM3A on TNBC bone metastasis., These findings serve as a critical theoretical foundation for the development of effective preventive and therapeutic strategies for TNBC, offering valuable insights for addressing this complex disease.

## Materials and methods

### Cell lines and cell culturing

The MCF-10 A mammary epithelial cell line and the human BC cell line MDA-MB-231 were obtained from Beijing Beina Chuanglian Institute of Biotechnology and used for in vivo experiments. The MDA-MB-231 cells were cultured in Dulbecco’s modified Eagle’s medium (DMEM-H) supplemented with 10% fetal bovine serum (FBS, Life Technologies), 0.25% trypsin, and penicillin-streptomycin (10,000 U) (Gibcao). The cells were maintained in a humidified incubator at 37 °C with 5% CO2.

### Generation of model cell lines

The lentiviral vectors GV248, Negative control virus CON077, and KDM3A knockdown LV-KDM3A-RNAi lentivirus were acquired from Shanghai Jikai Gene Company. LVpFU-GW-0077 and Ubi-MCS-3FLAG-CMV-EGFP were used to generate recombinant lentiviruses. The KDM3A targeting shRNA sequences was designed as follows: shRNA-1:5’-AAGCAACAACAATTCTGGTTT-3’; shRNA-2:5’-AAGCATTTAGATGAAAGCCAT-3’; shRNA-3:5’-AGGCAGCTGTACTCAGCCTAA-3’. Non-targeting control shRNA sequences used were: 5’- TTCTCCGAACGTGTCACGT − 3’. The Stable knockdown of KDM3A was confirmed through RT-PCR and Western blot analysis. The plasmid expressing sh-KDM3A was obtained from Shanghai Jikai Gene Company. Cell transfection was performed following the manufacturer’s protocol.

### RT-PCR

According to the manufacturer’s instructions, the total RNA was isolated from the cells using Trlzol reagent (Full gold). The concentration and purity of the total RNA were determined using the BC protein quantitative detection kit (Beijing Tiangen). Subsequently, cDNA was synthesized from 800 ng of RNA using a reverse transcription kit (Full gold). A real-time fluorescence measurement system (ABI7500, Thermo, USA) was used for data analysis. The primer sequences were as follows: KDM3A-F:5’-GGTTTCTCAGTCTGTCCGCA-3’; KDM3A-R:5’-TCTTGGTAACGTCGGTGTGG-3’; has β-Actin-F:5’-CATGTACGTTGCTATCCAGGC-3’; has β-Actin-R:5’-CTCCTTAATGTCACGCACGAT-3’; mouse actin-F:5’-GTGACGTTGACATCCGTAAAGA-3’; mouse actin-R:5’- GCCGGACTCATCGTACTCC-3’. The annealing temperature was set at 60 °C, and data analysis was performed using the 2-ΔΔCt method.

### Western blot analysis

Whole cell lysates were obtained using RIPA lysis buffer (Boster, AR0105-100). The samples were qualified using BC (Beijing full gold) quantitative determination. Proteins were separated on a 30% acrylamide gel (Amresco) and transferred to PVDF Transfer Membrane (Millipore). Subsequently, the membrane was blocked with 5% non-fat dry milk for 1 h at room temperature. After washing with TBST, the membrane was incubated overnight at 4 °C with the respective primary antibodies. The immunoblot was washed, diluted with TBST, and incubated at room temperature for 1 h. Finally, the protein bands were detected and photographed using a ChemiScope mini (Shanghai Qinxiang Scientific Instrument Co., Ltd.).

### Cell proliferation assay

A cell line with a robust growth status was selected, and a complete medium was prepared to obtain a single-cell suspension with a concentration of 5 × 10^4^ cells/mL. Subsequently, the cells were inoculated into a 96-well plate at a density of 5 × 10^3^ cells per well, and this process was repeated every 24 h. After 7 days, cell cultures were carefully aspirated, a 10% cell count kit-8(CCK-8) solution (Beijing Quanzhou Gold) was added, and incubated in an incubator at 37 °C. After 1 h, OD values were measured on a microplate reader at 450 nm.

### Cell migration and invasion assays

MDA-MB-231 cells at 90% confluence were selected, and the cell concentration was adjusted to 4 × 10^5^ cells/mL. In the lower chamber, 600 µL of complete medium was added and 100 µL of cell suspension was added to the upper chamber, with 3 replicate wells per group. The chamber was incubated at room temperature for 20 min after fixing the cells with 4% formaldehyde. Subsequently, the chamber was removed, and the cells were stained with Giemsa stain. Finally, 5 random fields were counted under a microscope.

### Cell cycle analysis and apoptosis detection

After 96 h of infection, the cells were trypsinized, centrifuged at 1000 r/min for 5 min, and resuspended in annexin V binding solution (BD company, USA) to prepare a single-cell suspension. PI/RNase staining solution (BD, USA) was added to the suspension, gently mixed, and incubated in the dark at 4 °C. Flow cytometry analysis was performed within 30 min.

### Hematoxylin and eosin (HE) staining

The Paraffin sections were deparaffinized in xylene and subsequently hydrated in alcohol. They were then counterstained with hematoxylin (3 min), differentiated with 1% hydrochloric acid alcohol for a brief period of 1–2 s, and rinsed with tap water to halt the differentiation process. To provide contrast staining, a 0.5% eosin ethanol solution was added for 1 min. The sections were then placed in 95% ethanol to remove any excess red color. After dehydration in xylene, the sections were carefully taken out and allowed to dry. Finally, they were sealed with neutral adhesive and observed under a microscope (Nikon Biomicroscope, E200).

### In vivo xenograft studies

Sixty female BALB/C nude mice, aged 4 weeks and from different litters, were randomly divided into control group and sh-KDM3A group, with 30 mice in each group. Subsequently, each group of nude mice was further divided into three subgroups, with 10 mice in each subgroup, based on the modeling time (10, 16, and 22 days). To establish a BC bone metastasis model, 1 × 10^6^ stable MDA-MB-231 cell lines, including those expressing (1) KDM3AshRNA, and (2) control empty vector, were resuspended in 200 µL of PBS/Matrigel and injected via tail vein. At the end of the experiment, the mice were euthanized using sodium pentobarbital. The tumors were carefully excised, promptly fixed in 15% formalin, embedded in paraffin, and stored appropriately for subsequent experiments. The tumor weights were also measured.

### IVIS, DR, and CT imaging

For imaging purposes, mice were injected with a fluorescein solution (150 mg/kg in PBS) 5 to 10 min before the imaging session. Prior to imaging, the mice were anesthetized with sodium pentobarbital. Imaging studies were conducted using a 64-slice helical Computed Tomography (CT)(Siemens), a Xenogen IVIS Spectrum (Perkin Elmer), and Digital Radiography (DR) (Philips).

The IVIS data were analyzed using Imaging J software, which included quantifying the light radiance emitted by the samples. The signal emitted by the light source was detected and characterized to extract relevant information.

### Statistical analysis

Data analysis was performed using SPSS 19.0 statistical software, allowing for comparison among multiple groups. One-way analysis of variance (ANOVA) was employed, and the t-test was used in cases where the variances were equal. The experimental data were presented as mean ± standard deviation (x ± s), and GraphPad Prism 8. 0 software was used for creating graphs.

## Results

### KDM3A is highly expressed in Breast cancer cells

**Fig. 1:**
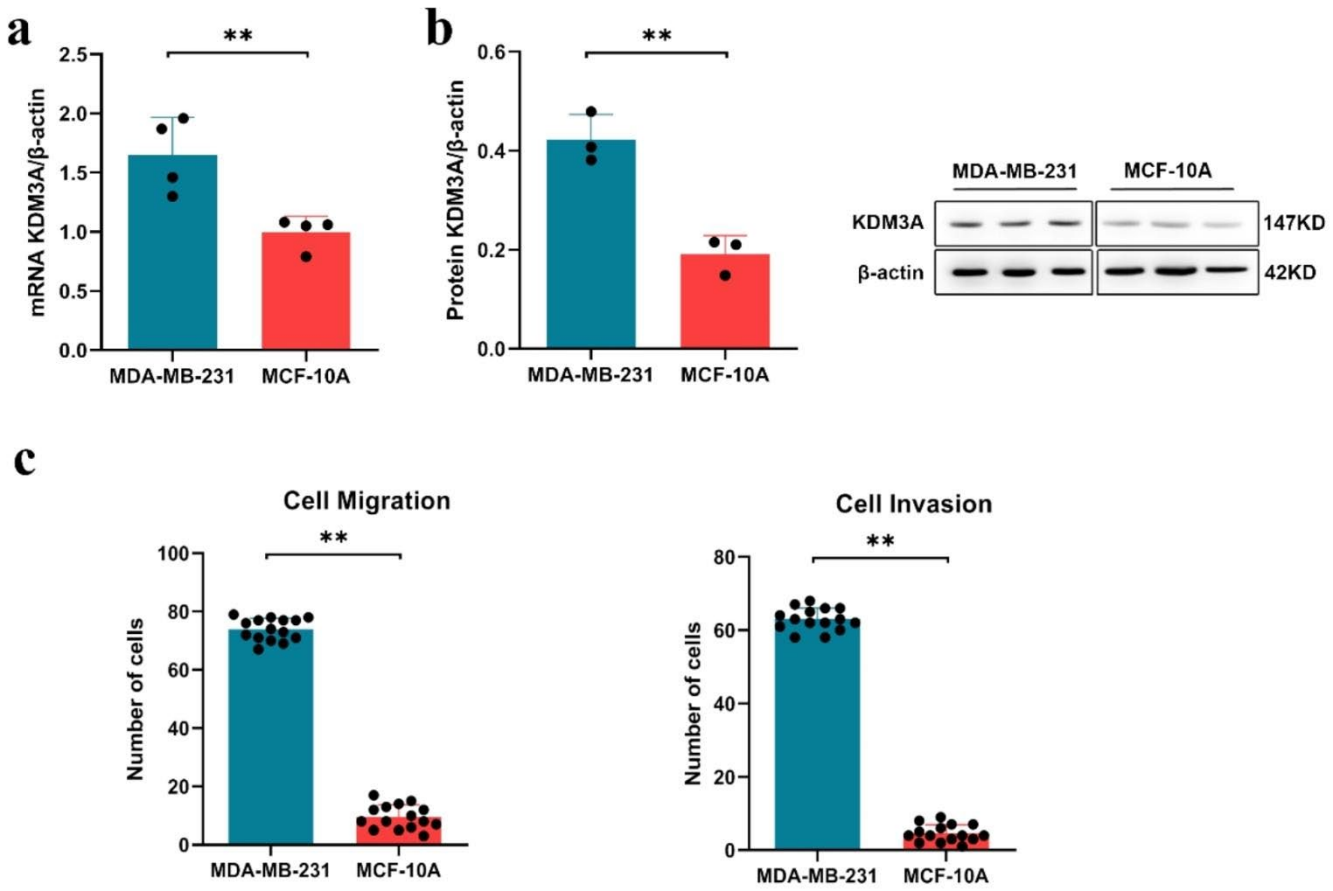
KMD3A is over-expressed in MDA-MB-231 cells. (**a**) The expression of KDM3A by RT-PCR; (**b**) Levels of KDM3A protein from breast cancer cell by Western blotting; (**c**) Transwell detection of invasive and migrating cell numbers of cells. Data are expressed as the mean ± SD; *P < 0.05, **P < 0.01

Through RT-PCR and Western blot analysis, we found that the mRNA and protein expression levels of KDM3A were significantly higher in BC MDA-MB-231 cells compared to normal breast epithelial MCF-10 A cells (*P* < 0.01, Fig. 1a, b). Furthermore, by conducting Transwell assays to analyze cell migration and invasion, we observed a significant increase in the migratory and invasive abilities of BC MDA-MB-231 cells compared to MCF-10 A cells (*P* < 0.01, Fig. 1c).

### Impact of KDM3A knockdown on the cell cycle and proliferation of TNBC cells

**Fig. 2:**
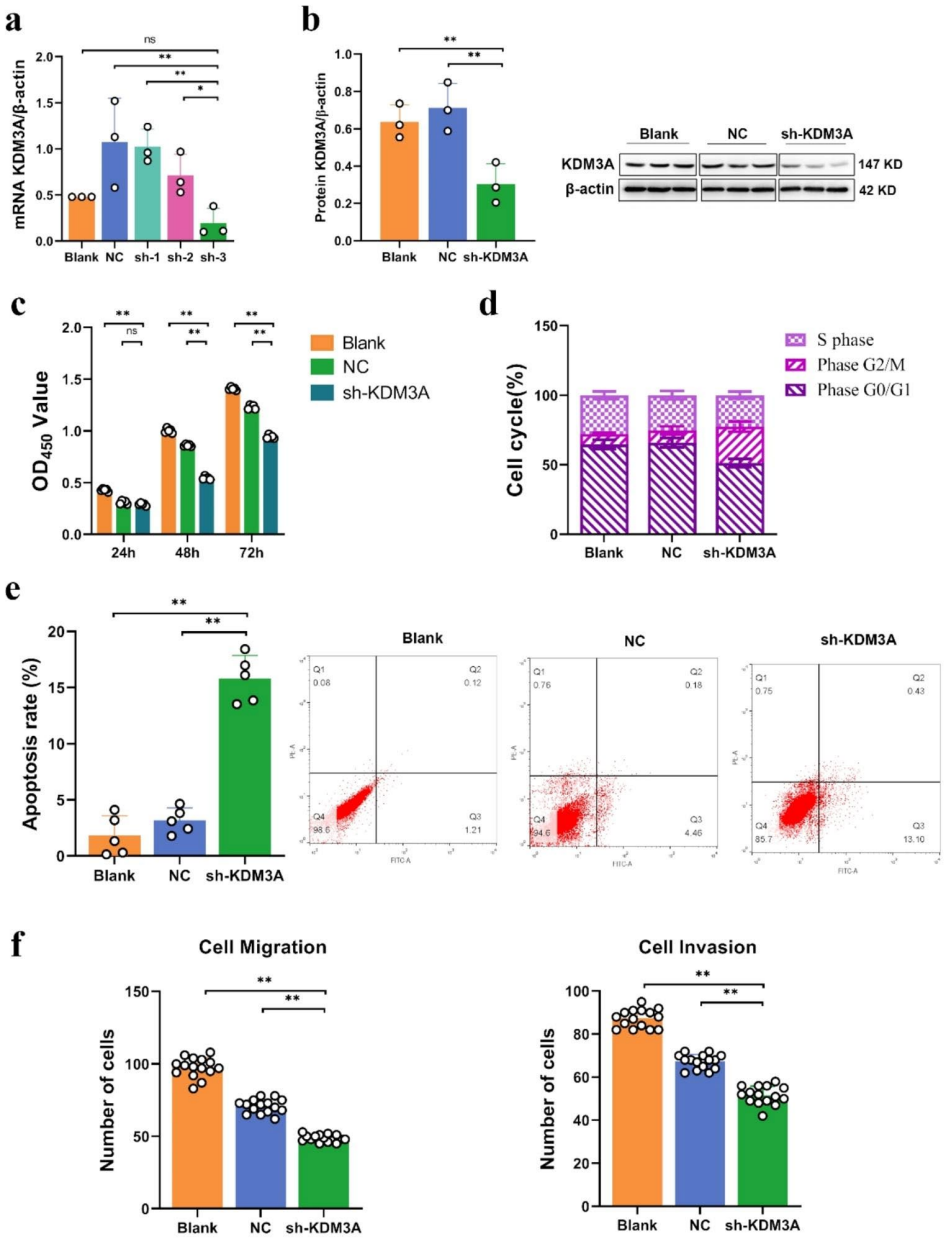
Knockdown of KDM3A expression in breast cancer cells suppress proliferation and metastasis. (**a**) Quantitative RT-PCR analysis of KDM3A expression levels after knockout by shRNA in MDA-MB-231 cells; (**b**) Levels of KDM3A protein from MDA-MB-231 cell by Western blotting; (**c**) Effect of KDM3A knockout on cell proliferation by CCK-8 detection; (**d**) Effect of KDM3A knockout on cell cycle detected by flow cytometry; (**e**) Detection of apoptosis by flow cytometry; (**f**) Effect of knockdown of KDM3A on cell invasion and migration by transwell detection. Data are expressed as the mean ± SD; *P < 0.05, **P < 0.01

In order to elucidate function role of KDM3A in the initiation and progression of BC, the expression of KDM3A in MDA-MB-231 cells was silenced by lentivirus transfection technique mediated by targeted short hairpin RNA (shRNA). The efficacy of KDM3A silencing was quantified via quantitative real-time polymerase chain reaction (qRT-PCR), and the sh-3 group exhibited the highest degree of KDM3A gene knockout (*P* < 0.05, Fig. 2a). Consequently, the sh-3 group was selected as the sh-KDM3A group for subsequent investigations. Subsequent Western blot analysis further confirmed a substantial reduction in KDM3A protein expression in the sh-KDM3A group (*P* < 0.05, Fig. 2b).

Having successfully established the KDM3A knockout model, we conducted a comprehensive analysis of its biological effects. The CCK-8 assay unveiled a substantial decline in optical density (OD) values over various time points in the sh-KDM3A group, indicating impaired proliferative potential compared to control cells (*P* < 0.01, Fig. 2c). Additionally, flow cytometry analysis revealed that, in comparison to the NC and blank groups, the sh-KDM3A group exhibited a significant decrease in the proportion of cells in the G0/G1 phase, while there was a significant increase in the proportion of cells in the G2/M phase (*P* > 0.05, Fig. 2d), with no change in the proportion of cells in the S phase. These findings indicate that silencing of the KDM3A gene led to cell cycle arrest in the G0/G1 phase. It is also noteworthy that the apoptosis rate of sh-KDM3A cells was significantly increased compared to other groups of cells (*P* < 0.01, Fig. 2e, f). Moreover, the transwell assay unequivocally demonstrated a remarkable reduction in the migratory and invasive capacities of the sh-KDM3A group (*P* < 0.01, Fig. 2f).

### Suppression of MDA-MB-231 cell growth in a nude mouse tumor transplantation model upon KDM3A knockdown

**Fig. 3:**
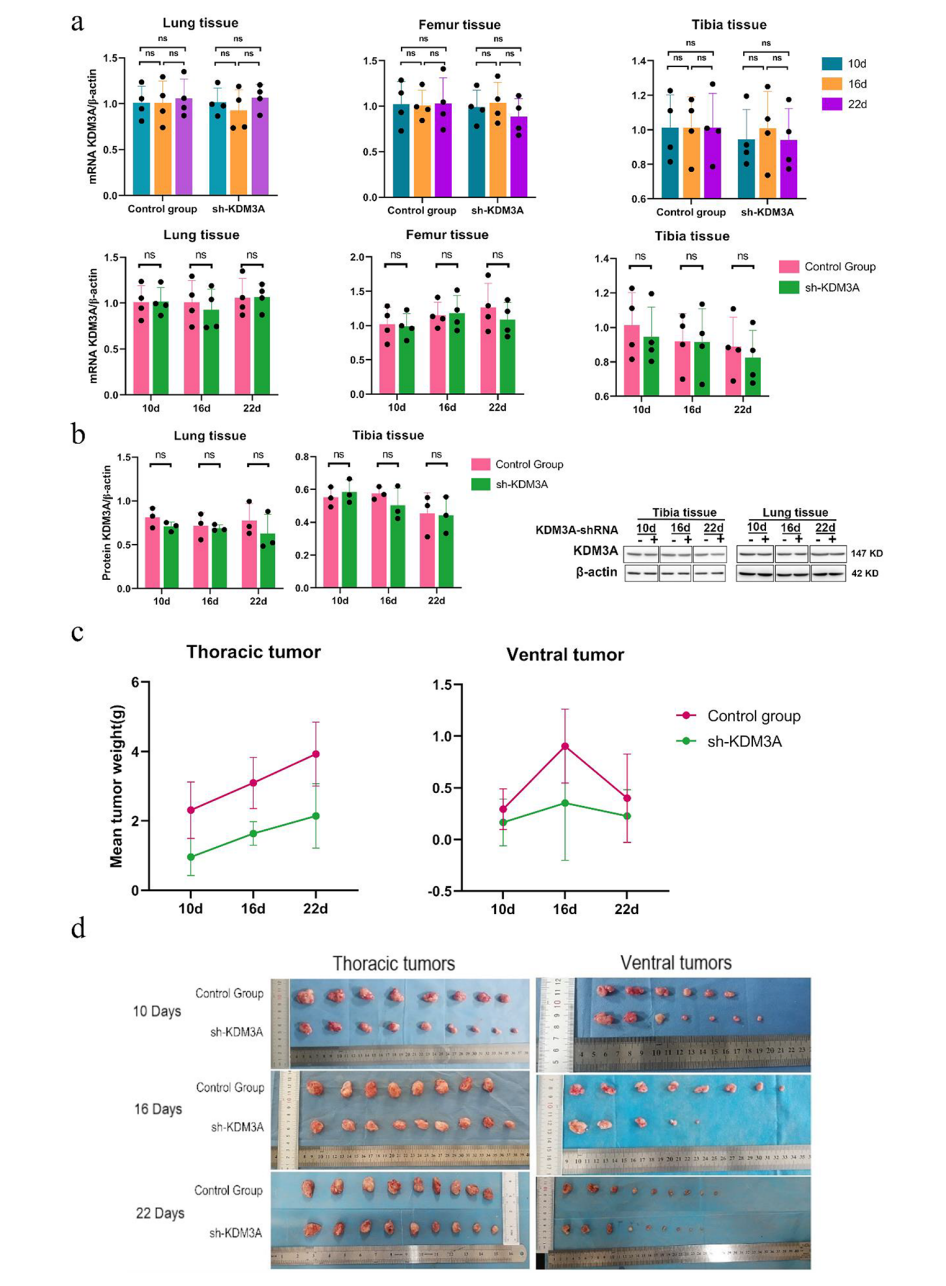
Knockdown of KDM3A inhibits KDM3A expression and the growth of breast cancer metastases. (**a**)Levels of KDM3A protein from different tissues by Western blotting; (**b**) Quantitative RT-PCR analysis of KDM3A expression levels at different times and in different tissues; (**c**) The tumors were removed and weighed after transplantation; (**d**) Pictures of transplanted tumors. Data are expressed as the mean ± SD; *P < 0.05, **P < 0.01

In vivo knockdown of KDM3A expression in metastatic BC was achieved through the utilization of MDA-MB-231 human BC nude mouse xenografts was to comprehensively evaluate the impact of KDM3A knockdown on metastatic BC Strikingly, the expression of the KDM3A gene in femur, tibia, and lung tissues exhibited no significant alterations in the difference of KDM3A gene expression in femur, tibia and lung tissues of rats in the sh-KDM3A group compared with the control group (*P* > 0.05, Fig. 3a); Western blot analysis of KDM3A protein expression at each time point in the sh-KDM3A group demonstrated not substantial deviation compared to the control group (*P* > 0.05, Fig. 3b).It is worth mentioning that in thoracic and abdominal tumors, the results showed that knockout of KDM3A inhibited tumor growth in the mouse model (*P* > 0.05, Fig. 3c,d).

**Fig. 4:**
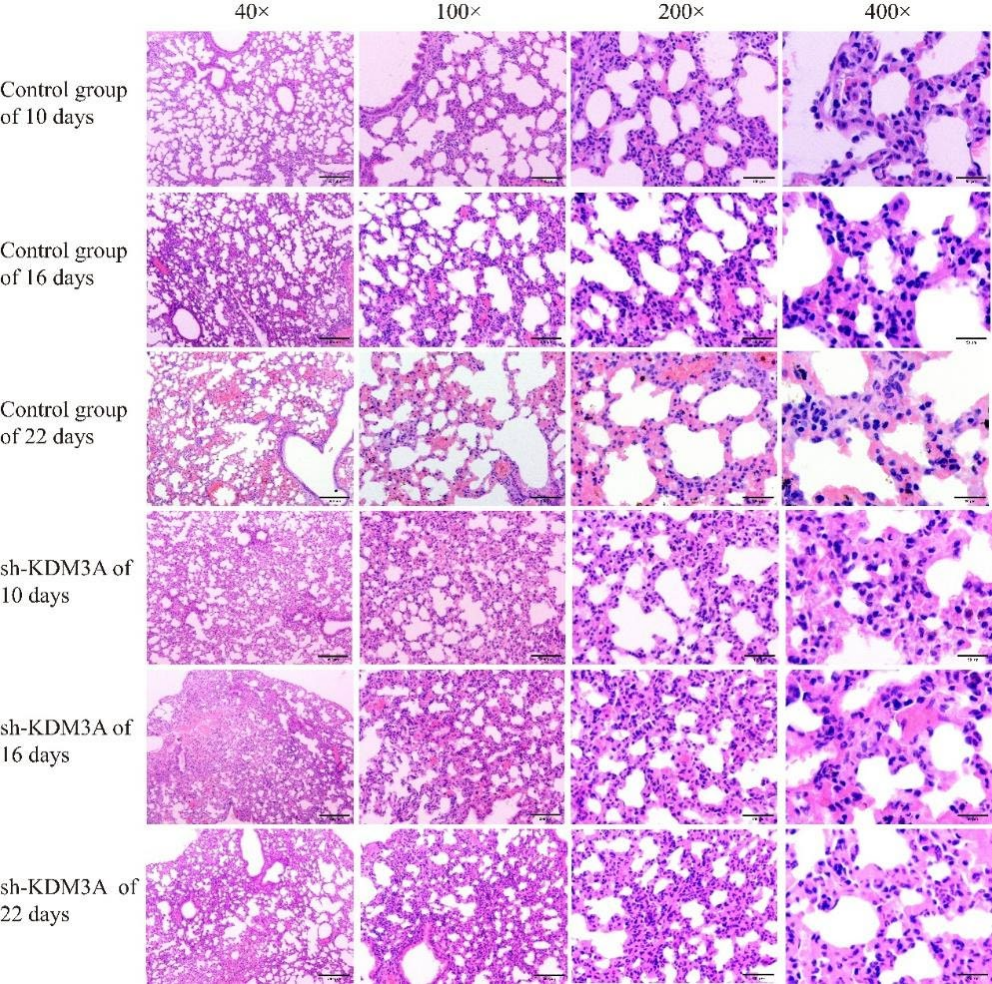
HE experimental results of lung tissue

**Fig. 5:**
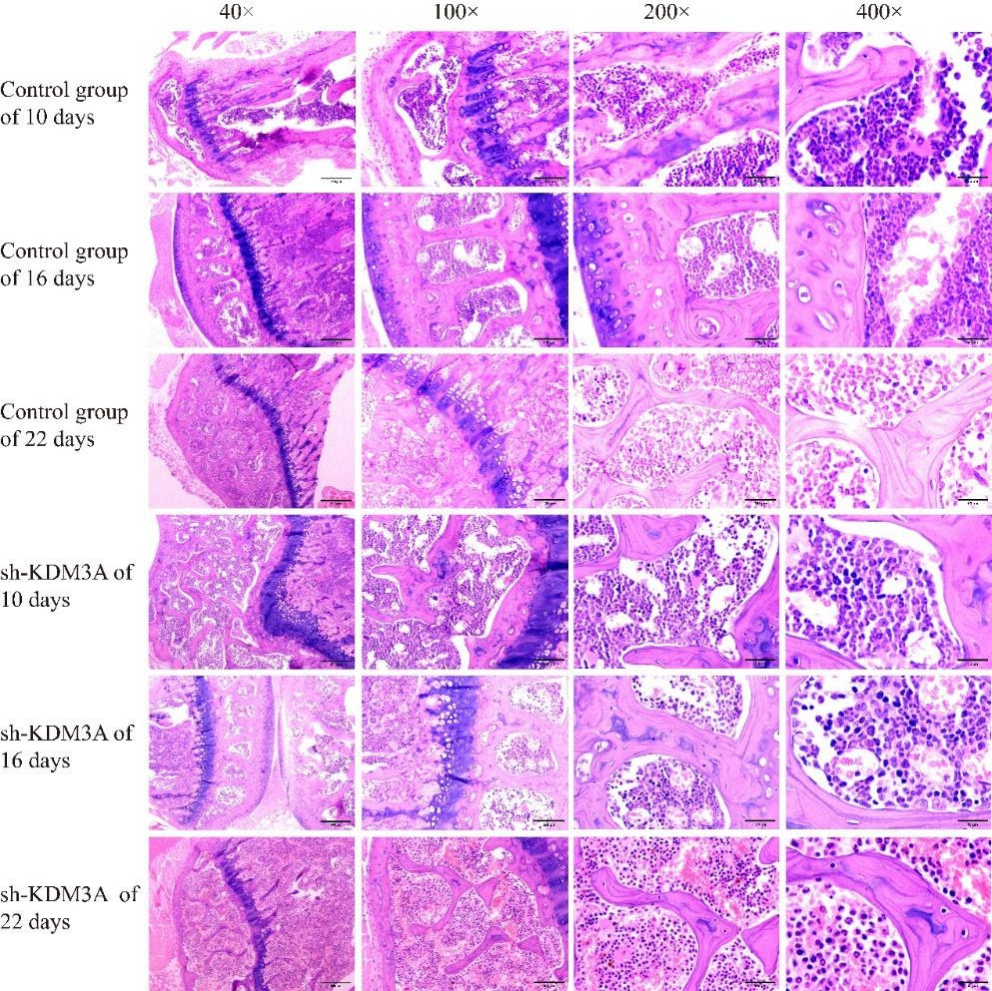
HE experimental results of tibia tissue

**Fig. 6:**
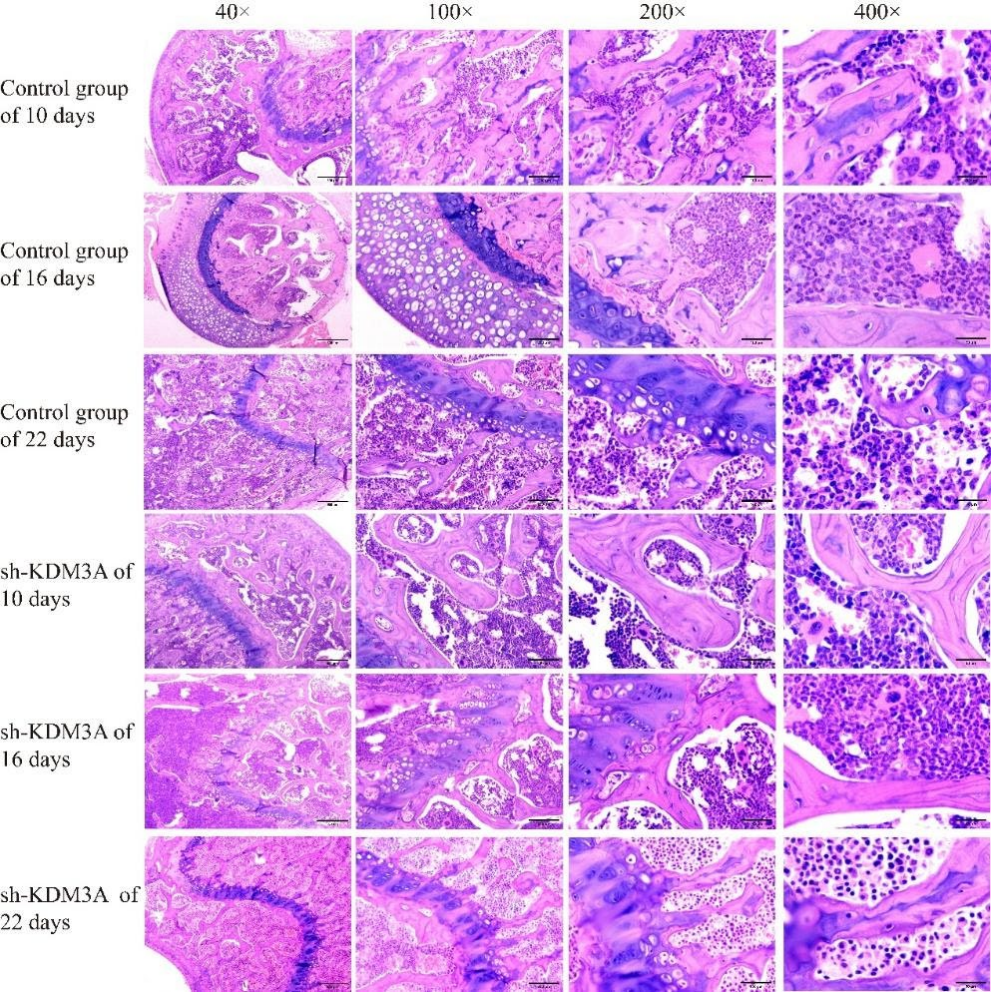
HE experimental results of femur tissue

After HE staining, the lung tissue structure was still normal in the sh-KDM3A group and the control group under microscopic approximation, with some alveolar walls broken and cystically dilated, some alveolar intervals thickened, more acute and chronic inflammatory cell infiltration, and some lung tissues congested and hemorrhagic; with the extension of time, the difference of lung tissue changes was not obvious; after 10 days, the lesions in the sh-KDM3A group were slightly improved compared with the control group (Fig. 4). Additionally, the general structure of tibia and femur tissue in sh-KDM3A and control group at different time periods were normal, and no obvious abnormality was found in bone marrow cavity and bone marrow tissue (Figs. 5 and 6).

**Fig. 7:**
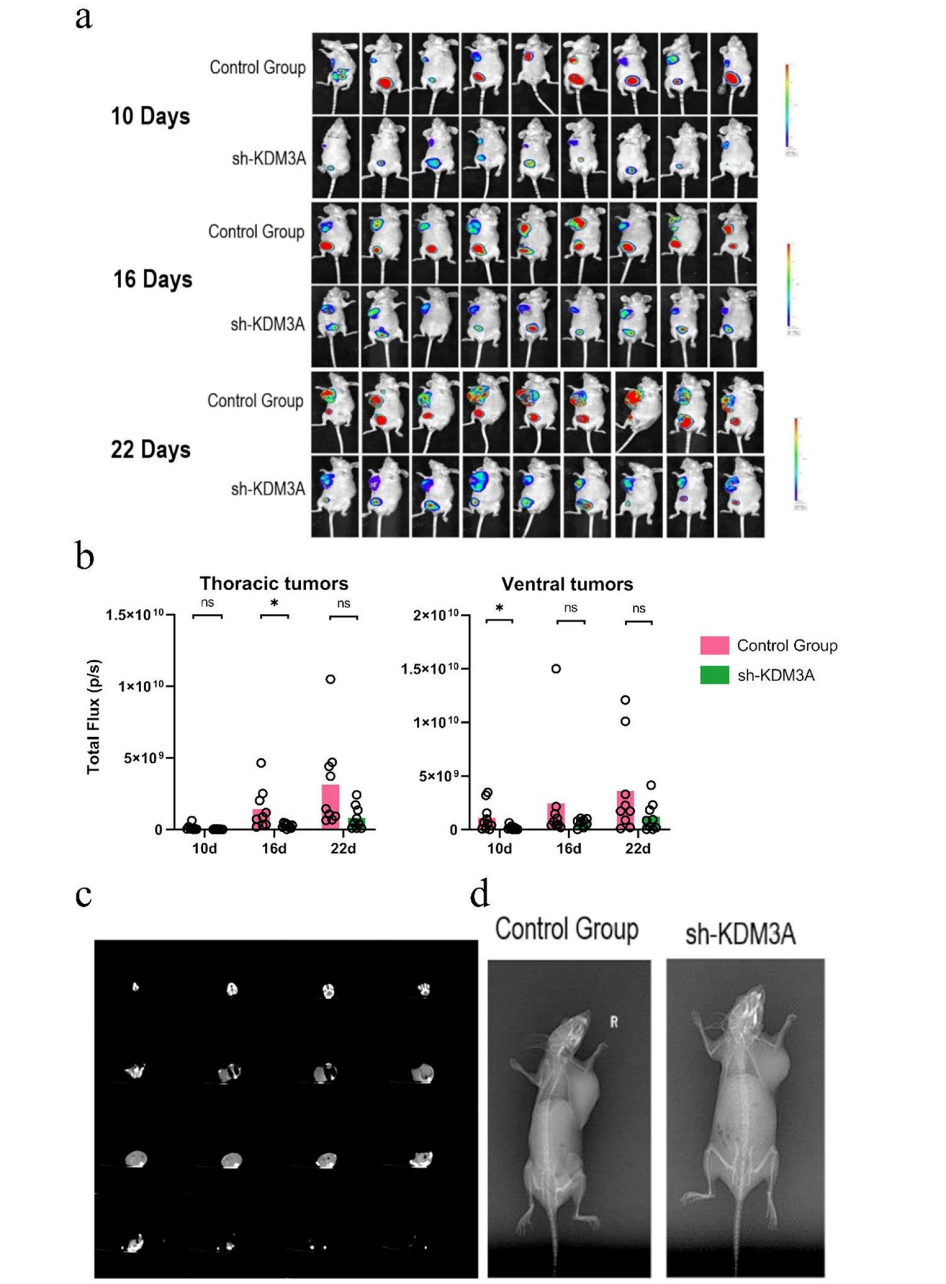
KDM3A expression reduces breast cancer metastasis. (**a**) In vivo fluorescence imaging of MDA-MB-231 cells in mice after KDM3A knockout; (**b**) Quantitative analysis of in vivo imaging fluorescence intensity; (**c**) Representative DR image of the control group and sh-KDM3A; (**d**) Representative CT images of the sh-KDM3A. Data are expressed as the mean ± SD; *P < 0.05, **P < 0.01

Intravital imaging using luciferase expression did not demonstrate a significant difference in luciferase expression in thoracic and abdominal tumor metastatic tissues between the sh-KDM3A group and the control group at various time points (*P* > 0.05, Fig. 7a, b). DR and CT imaging reveals no increase in BC malignancy in sh-KDM3A group compared to control group in BC bone metastasis model (Fig. 7c, d). All these findings are consistent with the in vitro results, indicating that knocking down KDM3A suppresses BC tumorigenesis and bone metastatic potential.

### Discuss

Metastasis is the leading cause of BC-related deaths globally (Man et al. 2023). KDM3A plays an important role in tumorigenesis and development (Li et al. 2018), Functioning as a crucial regulator of tumor growth and metastasis, KDM3A holds considerable promise as a potential target for anticancer therapies by impeding the activity of oncogenic transcription factors (Herzog et al. 2012; Jeon et al. 2022; Sui et al. 2021; Yoo et al. 2020). Moreover, it is significantly overexpressed in various cancers, including lung cancer (Liu et al. 2021), colorectal cancer (Sui et al. 2020), cervical cancer (Liu et al. 2020), Ewing’s sarcoma (Sechler et al. 2017; Wang and Quan 2021), bladder cancer (Wan et al. 2017), and ovarian cancer (Ramadoss et al. 2017b). The heightened expression of KDM3A in these diseases further supports its involvement in tumorigenesis and its correlation with unfavorable prognostic outcomes. Therefore, it is imperative to validate the biological function of KDM3A to elucidate its impact on BC metastasis mechanisms. In this study, we detected the KDM3A gene in MCF-10 A and MDA-MB-231 specimens, as this gene is significantly upregulated in cancer and correlates with poor prognosis in BC patients. Additionally, we found that in vivo knockdown of KDM3A suppressed cell viability, cell invasion, and bone metastasis.

In human infiltrative BC tissues, KDM3A exhibits a high expression level. Multiple studies have demonstrated that KDM3A deficiency effectively impedes the invasive growth of BC in both in vitro and in vivo settings (Berke et al. 2022; Ramadoss et al. 2017a). Remarkably, significant downregulation of KDM3A leads to the inhibition of cell proliferation, suppression of BC invasion, and reduced cell survival (Li et al. 2019). Additionally, KDM3A overexpression has been found to promote migration in BC cells, whereas KDM3A knockout results in migration inhibition (Zhao et al. 2016a). These findings strongly indicate the critical role of KDM3A knockdown in inhibiting BC cell proliferation and growth. Consistent with these observations, upon KDM3A knockout in MDA-MB-231 cells, a pronounced decrease in migratory and invasive capabilities and a significant increase in apoptosis rate were observed. Therefore, in this study, lentivirus-mediated knockdown of KDM3A using short hairpin RNA was employed, and fluorescence imaging and tumor weight measurements were evaluated in a mouse model of mammary tumors. Remarkably, KDM3A knockdown not only significantly inhibited tumor growth of MDA-MB-231 cells in vivo but also suppressed metastasis in nude mice, providing further support for the potential therapeutic targeting of KDM3A in TNBC. In addition, some scholars have proposed that the equivalent cell type and focal destruction of the envelope in myoepithelial cell layer or other epithelium-derived tissues are the most likely triggers for cancer invasion, and the influence of the surrounding structures of these epitheliums is largely ignored (Man et al. 2023), which may need to be taken into account and further analyzed.

There are studies suggesting that KDM3A plays an important role in estrogen receptor (ER) signal transduction. Specifically, KDM3A has implicated in ER signaling, as its knockdown results in the abolition of ER recruitment to the enhancer regions of ER target genes (Wade et al. 2015; Zhao et al. 2016b). Furthermore, KDM3A facilitates BC metastasis through its interaction with Brahma-related gene 1 (Sun et al. 2019). Moreover, under hypoxic conditions, KDM3A induces the expression of the transcriptional regulator slug, which positively regulates BC cell invasion (Ahn et al. 2020). Notably, KDM3A exhibits a dual role in BC, modulating both cell invasion and apoptosis through the demethylation of histone and non-histone p53 targets (Ramadoss et al. 2017a). To better understand the underlying mechanisms and the effects of KDM3A on various biological behaviors of BC cells, further analysis is needed. Nevertheless, contrary to our experimental results, KDM3A has been found to be downregulated in certain tumors. For example, low KDM3A expression is also associated with an aggressive phenotype and poor prognosis in gastric cancer (Ning et al. 2020). Furthermore, high KDM3A expression in BC patients demonstrated no significant association with prognosis compared to patients with low KDM3A expression (Yao et al. 2018). This indicates that KDM3A can exhibit both oncogenic and tumor suppressive properties, potentially depending on the specific cellular context and gene expression profiles regulated by KDM3A in different types of neoplasms. These distinct roles of KDM3A in different cancer types may be attributed to different signaling mechanisms, which necessitates further investigation.

In this study, the MDA-MB-231 cell line utilized belongs to the triple negative BC subtype. Although this cell line is widely used as a model for TNBC, it would be beneficial to include other BC cell lines representing different subtypes to provide a more comprehensive evaluation. Additionally, the robustness of the findings could be strengthened by validating the results using primary TNBC cells derived directly from patients, which would offer more relevant and clinically significant evidence.

In summary, the knockdown of KDM3A in the MDA-MB-231 cell line, a TNBC model, leads to enhanced apoptosis and suppressed proliferation, invasion, and migration. These findings collectively highlight the potential of KDM3As a therapeutic target for inhibiting the development and metastasis of TNBC. Furthermore, targeting KDM3A holds promise as a novel approach for the treatment of TNBC.

## Data Availability

The datasets used and/or analyzed during the current study are available from the corresponding author on reasonable request.
